# Protective Function of *Malus baccata* (L.) Borkh Methanol Extract against UVB/Hydrogen Peroxide-Induced Skin Aging via Inhibition of MAPK and NF-κB Signaling

**DOI:** 10.3390/plants11182368

**Published:** 2022-09-11

**Authors:** Chaoran Song, Chae Young Lee, Hwa Pyoung Lee, Mohammad Amjad Hossain, Zhiyun Zhang, Soo-Yong Kim, Minkyung Song, Jong-Hoon Kim, Jae Youl Cho

**Affiliations:** 1 Department of Integrative Biotechnology, College of Biotechnology and Bioengineering, Sungkyunkwan University, Suwon 16419, Korea; 2 College of Veterinary Medicine, Chonbuk National University, Iksan 54596, Korea; 3 State Key Laboratory of Systematic and Evolutionary Botany, Institute of Botany, The Chinese Academy of Sciences, Beijing 100093, China; 4 International Biological Material Research Center, Korea Research Institute of Bioscience and Biotechnology, Daejeon 34141, Korea

**Keywords:** *Malus baccata*, antioxidant, anti-inflammatory, aging

## Abstract

Ultraviolet (UV) irradiation induces ROS production, which activates activator protein (AP)-1 and nuclear factor (NF)-κB signaling and downstream molecules, ultimately triggering the generation of matrix metalloproteinases (MMPs) and degradation of collagen. The aim of this study was to investigate the protective effect of methanol extract from *Malus baccata* (L.) Borkh (Mb-ME) against aging. DPPH and ABTS assays showed that Mb-ME had a significant antioxidant capacity. Flow cytometry results indicated that Mb-ME attenuated UVB and H_2_O_2_-stimulated apoptosis and reactive oxygen species (ROS) generation. RT-PCR analysis in HaCaT and HDF cells suggested that Mb-ME treatment blocked the expression of MMPs, COX-2, IL-1β, IL-6, HYALs, and p53 while promoting the levels of TGM1, FLG, HASs, Sirt1, and Col1A1. Mechanically, Mb-ME inhibited the phosphorylation of MAP kinases and NF-κB signaling. Overall, these results strongly suggest that Mb-ME can be developed as an antiaging therapy.

## 1. Introduction

The skin is the largest organ that protects our body against damage including mechanical stimulation, physical damage, UV irradiation, and germ invasion [1]. The skin also regulates the immune system and prevents water loss. From the outside to inside, the skin is made up of the epidermis, dermis, and hypodermis [2]. The epidermis is mainly composed of keratinocytes while immune cells, fibroblasts, nerves, and hair follicles make up the dermis [3]. UV rays lead to a variety of skin diseases including cancer, degenerative aging, and inflammation [4]. The UVB spectra (290–320 nm) is the most damaging to the skin due to its shorter wavelengths and higher energy. The accumulation of excessive UVB exposure results in sunburn, oxidative stress, photoaging, DNA damage, and skin cancer [5,6].

Reactive oxygen species (ROS) include hydroxyl radicals, superoxide radicals, hydrogen peroxide, and peroxyl radicals such as O_2_^.^−, HO^.^, and H_2_O_2_ [7]. The balance of ROS in living organisms is controlled by its production and elimination. UVB affects mitochondrial electron transport, causing incomplete O_2_ reduction. Therefore, UVB disturbs the steady-state level of ROS in keratinocytes through inducing its generation. UVB damages the inner mitochondrial membrane structure and results in membrane potential collapse (ΔΨm) and subsequent outflow of cytochrome c [8]. Consequently, the released cytochrome c couples apoptotic protease activating factor-1 to trigger caspase 9 and then caspase 3 [9]. Activated caspases eventually cause the disintegration of apoptotic cells.

UVB-induced ROS triggers mitogen-activated protein kinases (MAPKs), JAK/STAT signaling pathways, nuclear factor (NF-κB), and Kelch-like ECH-associated protein 1-nuclear factor erythroid 2-related factor 2 (keap1-Nrf2-ARE), leading to the stimulation of transcription factors such as NF-κB and activator protein-1 (AP-1) [10]. The accumulation of ROS activates extracellular signal–regulated kinase (ERK), c-Jun amino-terminal kinase (JNK), p38, IκBα, IKKα/β, AKT, and other upstream molecules [11]. Ultimately, inflammation is exhibited by producing inflammatory mediators and proinflammatory cytokines including cyclooxygenease-2 (COX-2), interleukin-1β (IL-1β), and IL-6.

Upon UVB irradiation, matrix metalloproteinases (MMPs) are the enzymes responsible for the degradation of extracellular matrix (ECM) components in dermal tissue [12]. UVB promotes MMP activity and accelerates collagen degradation, leading to the loss of elasticity and formation of wrinkles. Moreover, hyaluronic acid (HA) is the main component in shaping skin structure and moisture [13]. HA is synthesized by HA synthases (HASs) at the inner plasma membrane [14]. Three different HAS (HAS-1, -2 and -3) isoforms are known to synthesize distinct molecular mass of HA. HA catabolism is achieved by hyaluronidases (HYALs) on the inner surface of the cell membrane. Thus far, six HYALs have been identified in humans. HYALs are expressed in different tissues and cleave various fragments of HA.

Melanin is produced by melanocytes in melanosomes and is a key element of skin pigmentation. Normal melanin synthesis protects the skin from UVB rays, whereas hyperpigmentation states are related to diseases, including LEOPARD syndrome, carney complex, and peutz-Jeghers syndrome [15]. Melanogenesis is an important process that is regulated by intrinsic and extrinsic factors. Among them, α-melanocyte stimulating hormone (α-MSH) is one of the most essential intrinsic factors stimulating melanogenesis [16]. α-MSH activates CREB, resulting in increased MITF and melanin production [17]. Tyrosinase and tyrosinase-related protein-1 (TYRP-1), located in the membrane of melanosomes, are critical melanogenic molecules that are transcriptionally mediated by MITF [18].

Accumulated evidence suggests the antioxidant, antimicrobial, and anticancer effects of natural plant extracts. *Malus baccata* (L.) Borkh is widely distributed throughout the world and cultivated across Asia and Europe. Its high levels of dietary fiber and polyphenols make it a potentially ideal food source for human nutrition. *M. baccata* extracts exhibited antiproliferative and proapoptotic effects to diverse cancer cells as well as antimicrobial activities [11]. However, the mechanism of the antioxidant and anti-aging functions of its methanol extract (Mb-ME) remains limited. Therefore, the aim of this study was to evaluate the antiaging activity of Mb-ME and explore its skin-protective functions in terms of antioxidant capacity, anti-apoptosis, moisturizing effect, and melanogenesis under UVB irradiation conditions.

## 2. Results

### 2.1. Antioxidant Effect of Mb-ME

**Figure 1:**
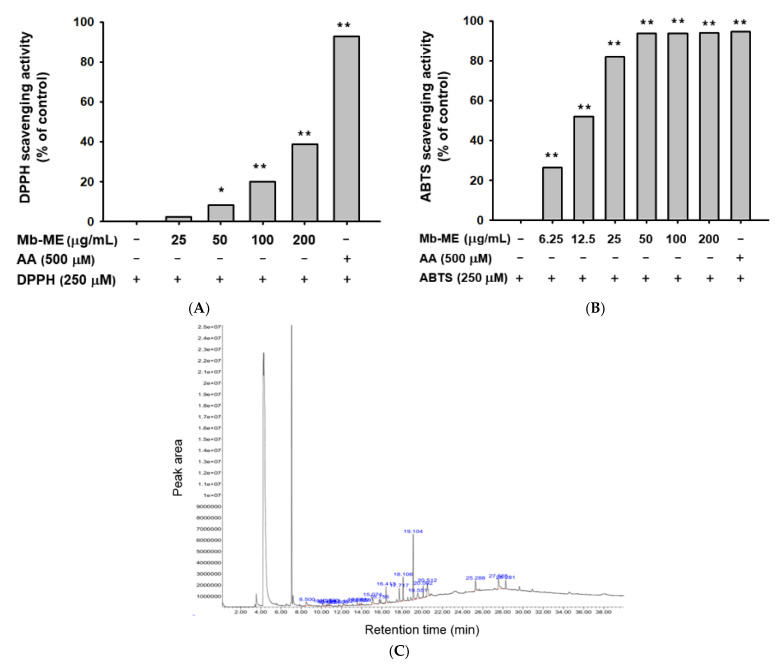
Antioxidant effect of Mb-ME and its phytochemical fingerprinting. (**A**,**B**) The antioxidant capacity of Mb-ME was measured by DPPH and ABTS assays. Ascorbic acid (AA) was used as a positive control. (**C**) Bioactive substances in Mb-ME were identified by gas chromatography–mass spectrometry. * *p* < 0.05 and ** *p* < 0.01 compared to the control group. −: No treatment and +: Treatment. Detailed information on retention time (R.T.) is also indicated in Table 1.

Among the well-developed methods that for estimating the free radical-scavenging activity, DPPH and ABTS assays are the most widely employed methods [19]. The capacity of antioxidants is achieved by measuring its scavenging ability to DPPH or ABTS generated in the aqueous phase. Because ascorbic acid (AA) is known to be an antioxidant and radical scavenger, we employed this compound as a positive control in DPPH and ABTS assays [20]. Mb-ME significantly scavenged DPPH and ABTS radicals with IC_50_ values of 241.6 μg/mL in the DPPH assay and 13.75 μg/mL in the ABTS assay, respectively (Figure 1A,B).

Mb-ME was further processed for the identification of bioactive compounds by GC-MS analysis. In Figure 1C, benzofuran (19.104 min), hydroquinone (20.512 min), benzenepropanoic acid (27.565 min), phenol (15.074 min), and other bioactive components were identified, suggesting that the antioxidant capacity of Mb-ME may be attributed to these components. Other compounds identified in Mb-ME were listed in Table 1.

### 2.2. Anti-Photoaging Effect of Mb-ME against UVB Irradiation in HaCaT Cells

**Figure 2:**
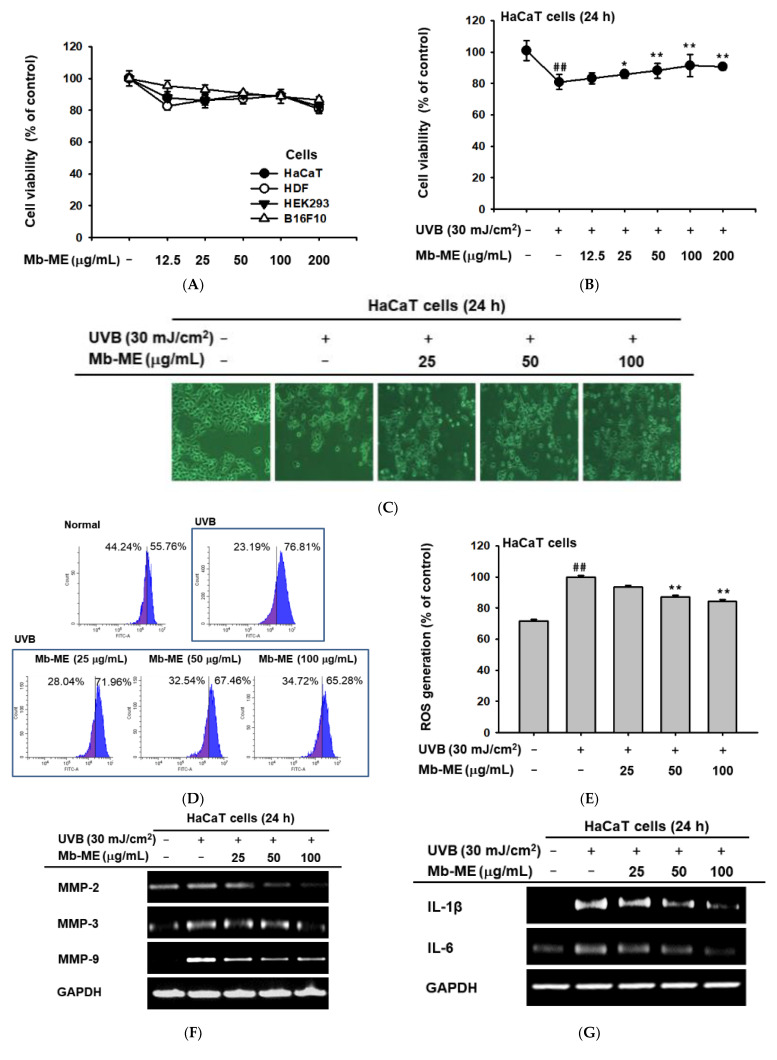
The protective function of Mb-ME upon UVB irradiation. (**A**,**B**) HaCaT, HDF, and HEK293 cells were treated with Mb-ME for 24 h, while B16F10 cells were incubated with Mb-ME for 48 h with or without UVB exposure. The cytotoxicity or cell viability of Mb-ME was then determined by an MTT assay. (**C**) The images of the HaCaT cells treated with UVB with or without Mb-ME were obtained. (**D**,**E**) Flow cytometry was conducted to explore the function of Mb-ME on ROS generation. (**F**,**G**) The HaCaT cells were irradiated with UVB in the absence or presence of Mb-ME and subjected to RT-PCR. The mRNA expression of MMP-2, 3, 9, IL-1β, and IL-6 was tested. * *p* < 0.05 and ^##^ *p* < 0.01 compared to the normal group. ** *p* < 0.01 compared to the control group. −: No treatment and +: Treatment.

The likelihood of a drug being developed depends first and foremost on its cytotoxicity. Therefore, the cytotoxicity of Mb-ME was evaluated by MTT assay. As shown in Figure 2A, Mb-ME was not cytotoxic at concentration up to 200 μg/mL in all tested cell lines. Whether Mb-ME can protect against cell death from UVB irradiation was further investigated by MTT assay. As shown in Figure 2B, compared with normal group, UVB irradiation significantly induced 19.1% cell death. Meanwhile, compared to UVB-induced group, Mb-ME treatment recovered cell viability in a concentration-dependent manner. Subsequently, we studied the morphology of UVB-exposed and Mb-ME-treated HaCaT cells by phase-contrast microscopy. UVB irradiation caused morphological changes such as cell shrinkage, resulting in damaged cytoskeletons and floating round dead cells (Figure 2C). Mb-ME suppressed these UVB-induced morphological changes. In addition, to determine whether ROS plays a major role as primary signaling molecules in the UVB-provoked photoaging response, DCF-DA staining and flow cytometry were applied. HaCaT cells were exposed to UVB and Mb-ME for 24 h. Compared to normal group (non-UVB irradiation group), UVB markedly elevated ROS production. The UVB-stimulated ROS levels in control group were reduced by Mb-ME at 50 and 100 μg/mL (Figure 2D,E). Since UVB-induced MMP secretion is a sign of skin photoaging, we investigated the effects of Mb-ME on UVB-activated MMPs expression. As expected, a marked increase of MMP-2, 3, and 9 was observed after cells were irradiated with UVB (Figure 2F). These increases were largely concentration-dependently blocked by Mb-ME. On the other hand, as shown in Figure 2G, UVB exposure causes skin inflammation whereas Mb-ME significantly decreased mRNA expression of IL-1β and IL-6.

### 2.3. Effect of Mb-ME on H_2_O_2_-Induced Damage in HaCaT Cells

**Figure 3:**
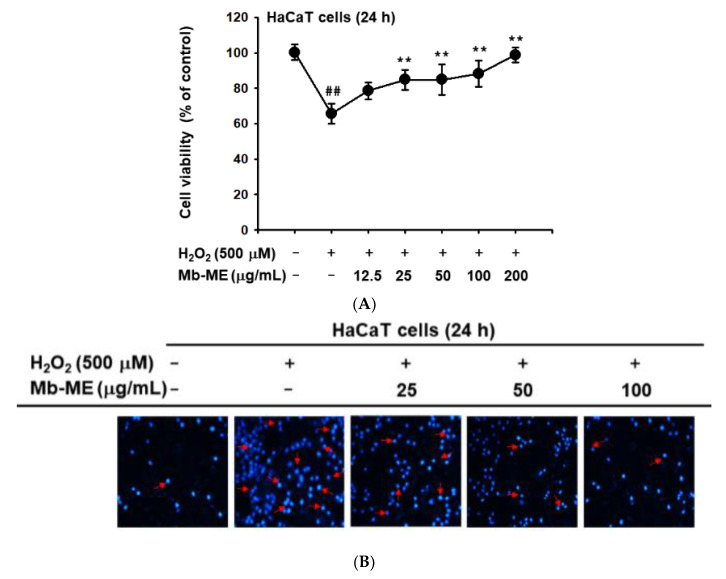

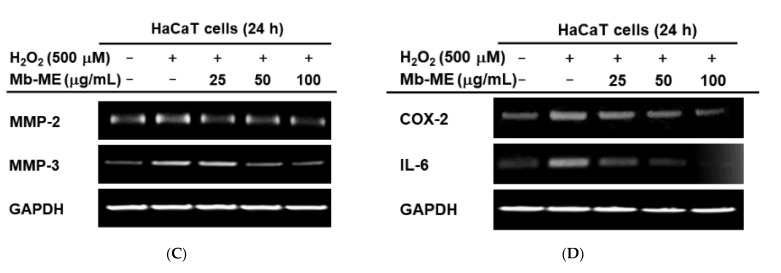
The antioxidant effect of Mb-ME against H_2_O_2_-induced damage in HaCaT cells. (**A**) The effect of Mb-ME on HaCaT cell viability was measured by MTT after H_2_O_2_ stimulation. (**B**) DAPI staining in HaCaT cells treated with UVB or Mb-ME. (**C**,**D**) The effect of Mb-ME on mRNA expression of MMP-2, 3, COX-2, and IL-6 was determined by RT-PCR. ^##^ *p* < 0.01 compared to the normal group. ** *p* < 0.01 compared to the control group. −: No treatment and +: Treatment.

MTT assay revealed H_2_O_2_ treatment markedly downregulated 34.4% cell viability in HaCaT cells while it was distinctly upregulated by Mb-ME (Figure 3A). During apoptosis, chromatin is converted to an inert, highly concentrated form, making it one of the criteria for identifying apoptosis [21]. DAPI staining revealed that H_2_O_2_ greatly exacerbated nuclear DNA condensation compared with the control (Figure 3B). However, Mb-ME treatment significantly alleviated DNA damage (Figure 3B). Similar to UVB induction, H_2_O_2_ treatment increased MMP secretion and IL-6 expression in control group without treating Mb-ME, compared to non-UVB-treated normal group. Mb-ME reduced the levels of MMP-2, MMP-3, COX-2, and IL-6 (Figure 3C,D), indicating that Mb-ME diminished H_2_O_2_-induced chronic skin inflammation and senescence.

### 2.4. Mb-ME Promoted Moisture Retention and Collagen Synthesis

**Figure 4:**
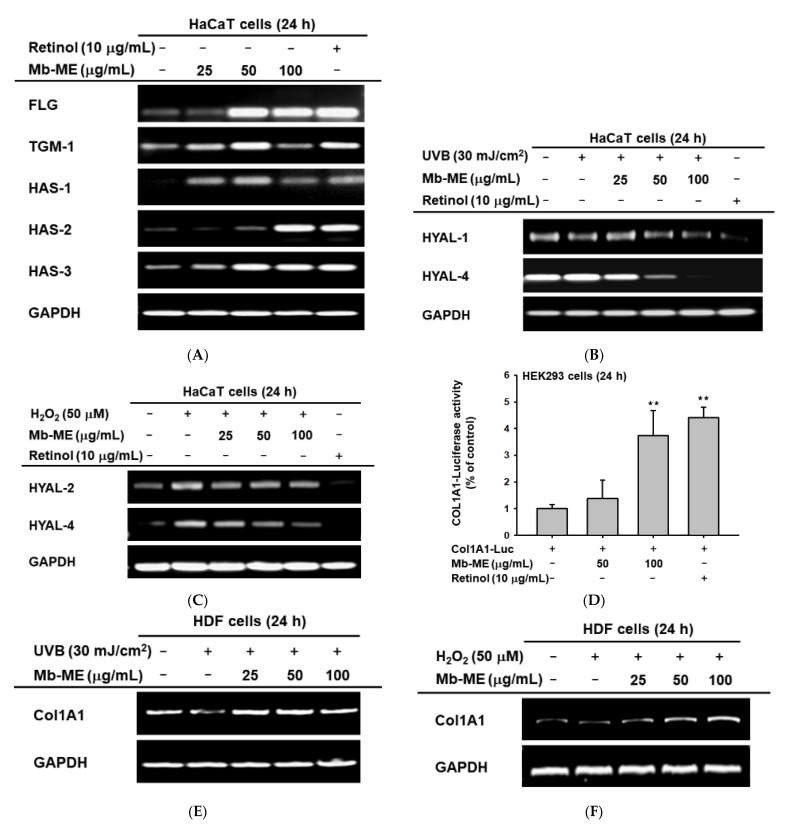
The effect of Mb-ME on moisture retention and collagen synthesis. (**A**) The mRNA levels of moisture-related genes in Mb-ME or retinol treated HaCaT cells was examined. (**B**,**C**) The effect of Mb-ME on expression of HYALs under UVB or H_2_O_2_ stimulation was studied by RT-PCR. (**D**) Luciferase activity in HEK293 cells was detected after transfection of COL1A1-Luc and treatment of Mb-ME. (**E**,**F**) Upon UVB or H_2_O_2_ induction, the mRNA expression level of collagen 1 in Mb-ME-treated HDF cells was determined. ** *p* < 0.01 compared to the normal group. −: No treatment and +: Treatment.

FLG and TGM1 are key proteins for skin barrier structure and function. Hyaluronic acid (HA) is one of the main components of the extracellular matrix, which is synthesized by three hyaluronan synthases (HAS-1, HAS-2, and HAS-3). Retinol has been reported to suppress MMPs and gelatinase generation, and to promote expression of collagen and moisturizing factors in photoaged skin [22,23]. Additionally, clinical trial has shown that retinol reduces the formation of fine wrinkles [24]. Therefore, we chose this compound as a positive control in this experiment. We found that Mb-ME significantly augmented the levels of FLG, TGM-1, HAS-1, HAS-2, and HAS-3 (Figure 4A). Furthermore, the expression of HYAL-1 and HYAL-4 were investigated by RT-PCR after UVB exposure. Our results indicated that Mb-ME decreased HYAL-1 and HYAL-4 expression (Figure 4B). Simultaneously, the degradation of HA by HYAL-2 and HYAL-4 was activated in the presence of hydrogen peroxide, which was inhibited by Mb-ME in a dose-dependent manner (Figure 4C). In addition, Mb-ME (100 μg/mL) enhanced Col1A1-mediated luciferase activity in transfected human kidney cell line (HEK293 cells) (Figure 4D). Consistent results were obtained through the measurement of Col1A1 by RT-PCR. As expected, UVB and H_2_O_2_ lowered Col1A1 expression whereas Mb-ME prevented UVB-induced degradation of collagen I (Figure 4E,F).

### 2.5. Regulatory Mechanisms of Mb-ME on UVB-Mediated HaCaT Cells

**Figure 5:**
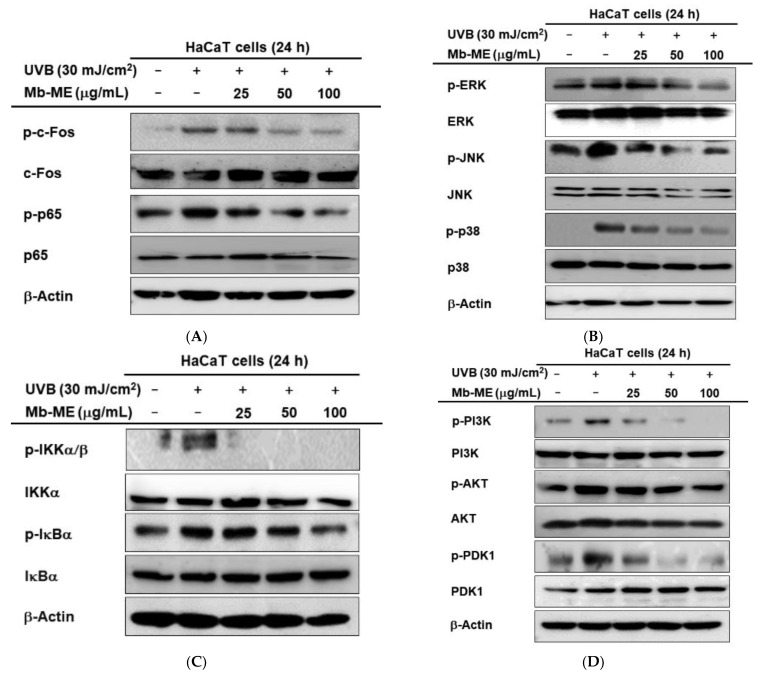

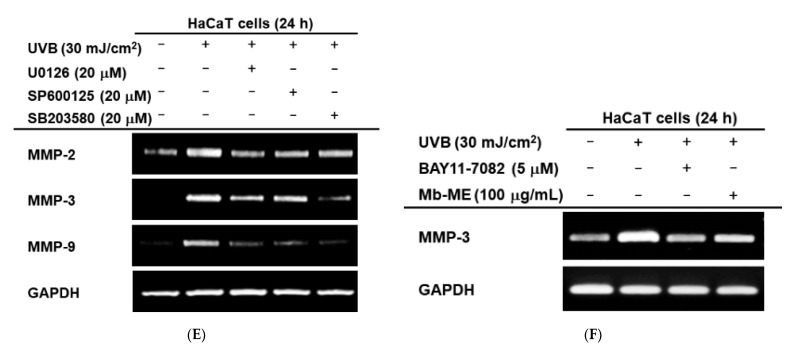
The regulatory mechanisms of Mb-ME on UVB-mediated HaCaT cells. (**A**–**D**) The HaCaT cells were stimulated with UVB with or without Mb-ME and were subjected to immunoblotting. (**E**,**F**) Following induction UVB, HaCaT cells were treated with specific inhibitors of MAPKs or NF-κB. RT-PCR was performed to measure MMP-2, 3, and 9. −: No treatment and +: Treatment.

In keratinocytes, previous studies have shown that ultraviolet light activates the NF-κB and AP-1 signaling pathways that are essential transcription factor releasing pro-inflammatory cytokines [25]. We first examined the effect of Mb-ME on the phosphorylation level of c-Fos and p65, the subunits of AP-1 and NF-κB, respectively. As shown in Figure 5A, phosphorylated c-Fos and p65 were stimulated by UVB and attenuated by Mb-ME. Next, we tested the effect of Mb-ME on AP-1 upstream signal proteins. As shown by western blot analysis (Figure 5B), UVB irradiation induced a prominent increase in phosphorylated MAPK proteins, including ERK, JNK, and p38. The treatment of HaCaT cells with Mb-ME resulted in an obvious inhibition in UVB-induced increase in the phosphorylation of MAPK proteins. Meanwhile, UVB irradiation significantly increased the phosphorylation levels of IκBα and IKKα/β proteins (Figure 5C). Phospho-AKT, PDK1, and PI3K, the upstream molecules of NF-κB, were also enhanced by UVB treatment while they were suppressed by Mb-ME (Figure 5D).

These results were proven by determining the expression levels of MMP-2, MMP-3, and MMP-9 using MAPK inhibitors upon UVB irradiation. These inhibitors suppressed the expression of MMPs in varying degrees (Figure 5E). U0126 (ERK inhibitor) particularly reduced the expression of MMP-2 while SB203580 (p38 inhibitor) blocked the mRNA expressions of MMP-3 and MMP-9. SP600125 (JNK inhibitor) slightly downregulated MMP-2 and MMP-3, but strongly decreased MMP-9. Meanwhile, Mb-ME reduced UVB-induced MMP-3 expression to the similar extent as BAY11-7082, an NF-κB inhibitor (Figure 5F). The inhibitor results suggested that MAPK and NF-κB are involved in UVB-mediated ECM degradation.

### 2.6. Anti-Apoptotic Effects of Mb-ME in HaCaT Cells

**Figure 6:**
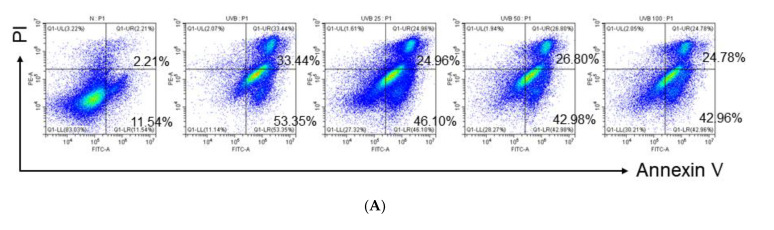

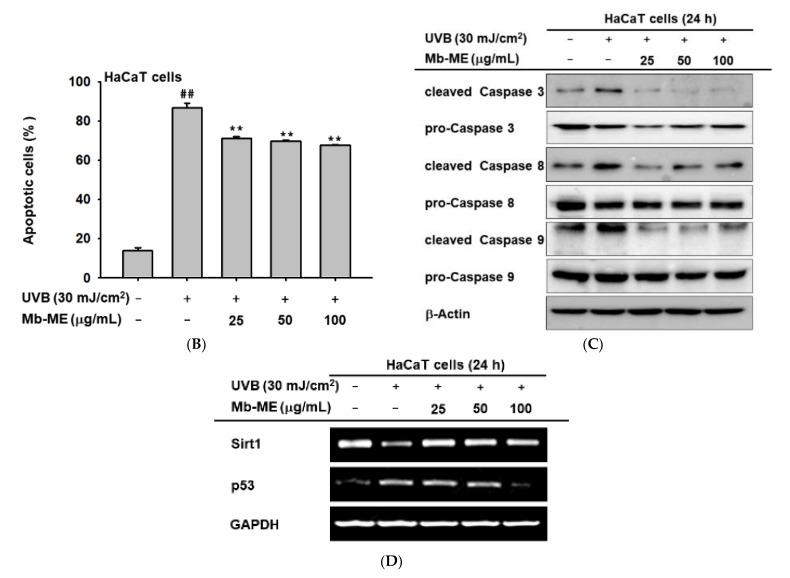
The effects of Mb-ME on apoptosis. (**A**,**B**) Under UVB irradiation, PI and Annexin V-FITC staining in HaCaT cells was carried out to determine the role of Mb-ME in apoptosis. (**C**) UVB and Mb-ME treated cells were subjected to immunoblotting, and Caspase-related proteins were examined. (**D**) The mRNA expression of Sirt1 and p53 was tested. ^##^ *p* < 0.01 compared to normal group. ** *p* < 0.01 compared to control group. −: No treatment and +: Treatment. No ^##^ or ** indicates not significant.

Extensive studies have revealed that UVB radiation leads to irreversible DNA damage, eventually leading to apoptosis. Flow cytometry was employed to evaluate the anti-apoptotic potential of Mb-ME by using propidium iodide (PI)-annexin V staining. Compared to untreated normal group, UVB dramatically triggered apoptosis in control group (Figure 6A,B). The enhancement was observably diminished by Mb-ME (Figure 6A,B). Caspases, one of the hallmarks of apoptosis, were determined by Western blot. Effect of Mb-ME on caspases was evaluated by the ability of Mb-ME to alter the inactive and active forms of caspases in HaCaT cells. As shown in Figure 6C, cleaved caspases 3, 8 and 9 were activated by UVB while attenuated by Mb-ME. It is widely reported that Sirt1 confers protection against UVB-damaged apoptosis in skin keratinocytes. Consistent with previous studies, UVB exposure significantly blocked Sirt1 expression whereas Mb-ME sharply recovered the mRNA expression of Sirt1 (Figure 6D). In addition, the activation of p53 by UVB was inhibited by Mb-ME in a concentrated manner (Figure 6D).

### 2.7. Effect of Mb-ME on Melanogenesis

**Figure 7:**
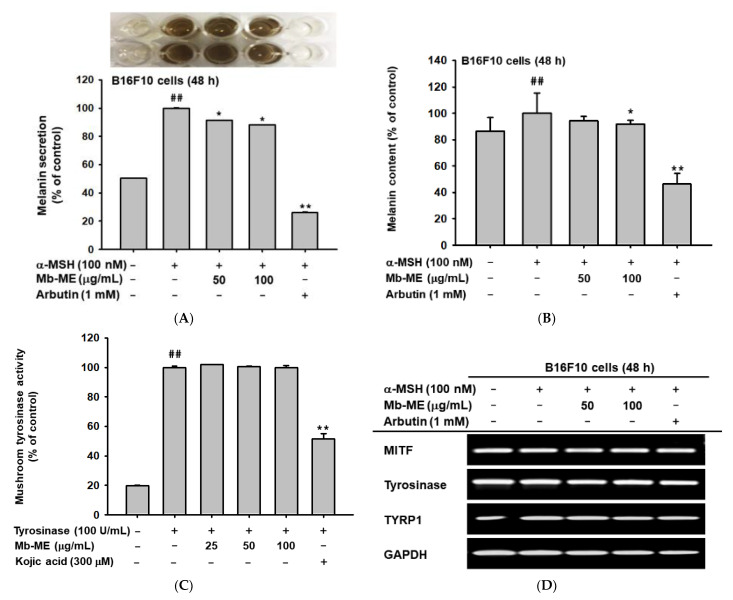
The effects of Mb-ME on melanogenesis. (**A**,**B**) The effect of Mb-ME on melanin secretion and content was investigated in B16F10 cells. (**C**) Tyrosinase activity was detected with or without Mb-ME. (**D**) Mb-ME, α-MSH, or arbutin (positive control) were used to treat B16F10 cells. The mRNA expression of MITF, TYRP1, and tyrosinase was measured. ^##^ *p* < 0.01 compared to normal group. * *p* < 0.05 and ** *p* < 0.01 compared to control group. −: No treatment and +: Treatment. No ^##^ or ** indicates not significant.

Melanin is secreted by melanocytes to protect the skin from UV damage through broadband ultraviolet adsorption. As shown in Figure 2A, Mb-ME did not reduce the cell viability of B16F10 cells. To investigate the effect of Mb-ME on melanin secretion, B16F10 cells were induced with α-MSH in the absence or presence of Mb-ME. Mb-ME significantly suppressed melanin secretion at 50 and 100 μg/mL concentrations, while Mb-ME only inhibited melanin content at 100 μg/mL (Figure 7A,B). Since tyrosinase is a rate-limiting enzyme that regulates melanin production, we next tested whether the inhibitory effect of Mb-ME on melanin secretion and contents involved tyrosinase or not. Intriguingly, Mb-ME did not affect mushroom tyrosinase activities (Figure 7C). Consistently, RT-PCR results confirmed this finding by determining the mRNA expression of tyrosinase. The expression of MITF and TYRP1, the essential regulators in melanin formation, was also not altered by Mb-ME (Figure 7D).

## 3. Discussion

As the largest organ of our body, the skin protects us against UV radiation, moisture loss, microbes, and other hazardous elements. Excessive and prolonged skin exposure to UVB causes oxidant stress, inflammation, and ROS hyperactivation [26,27]. The main goal of skin care is to prevent or slow down the process of skin aging with minimal adverse effects. Botanicals with antioxidant and immunomodulatory properties are expected to be developed as treatments for a variety of skin conditions including aging. In this study, we examined the role of Mb-ME on apoptosis, skin aging, melanogenesis, and antioxidant capacity as well as its mechanism. Mb-ME was shown to exhibit antioxidant, anti-apoptosis abilities while mitigating ROS generation, skin inflammation, and loss of collagen through the MAPK and NF-κB pathways.

We found Mb-ME promisingly dose-dependently enhanced DPPH and ABTS-radical scavenging activities (Figure 1A,B). These results indicate that Mb-ME has antioxidant capacity. Previous reports have shown that the antioxidant capacity measured by various methods is different. The antioxidant capacity of plant extracts is related to the type of ingredients contained in the extract and extractant. The correlation between ABTS and total phenolics was stronger than DPPH [28]. ABTS assay is suitable for hydrophilic and lipophilic systems, while DPPH assay is suitable for hydrophobic systems [29,30]. Since Mb-ME is a methanol extract, fat-soluble antioxidants may not be sufficiently extracted. In fact, our results showed that IC_50_ of DPPH assay is much lower than ABTS test. Mb-ME was further processed for the identification of bioactive compounds by GC-MS analysis. It was identified as containing diverse bioactive substances such as benzofuran, hydroquinone, benzenepropanoic acid, and phenol (Figure 1C). Among them, benzofurans figure in three principal fields in the area of cosmetics, antiaging, and depigmentation as well as UV absorbers for sunscreen [31,32]. It has been reported that hydroquinone inhibits tyrosinase, reduces the number of melanocytes, and interferes with melanin production [33]. Therefore, it is often used in lightening creams, moisturizers, and serums. According to our results, the above components are the main anti-aging ingredients in Mb-ME.

UVB stimulates MMPs expression, which is a hallmark of skin aging. MMPs degrades an extremely broad array of ECM compositions and collagen in connective tissues, thereby triggering skin wrinkling [34]. With aging, the level of MMPs increases with decreasing collagen synthesis. Consistent with these reports, MMP-2, -3, and -9 levels increased in our condition, while Col1A1 was declined in keratinocytes and HDF cells after UVB or H_2_O_2_ induction (Figure 4). RT-PCR data revealed that Mb-ME hindered stimulation caused by UVB and H_2_O_2_ and elevated Col1A1 mRNA expression. Col1A1 encodes type I collagen, a fibrillar collagen present in lots of connective tissues [35]. Collagen is an important scaffold protein that makes skin smooth and elastic [36]. Therefore, the degradation of ECM caused by aging is alleviated by Mb-ME, and skin elasticity is improved. Meanwhile, as reported, UVB activates the generation of inflammatory mediators, leading to an increase in chronic inflammation and inflammatory aging [37]. This is verified by comparing the aforementioned to HaCaT cells without UVB treatment (Figure 2B,C). UVB-induced elevated expressions of IL-1β, IL-6, and COX-2 were abrogated by Mb-ME. This indicates that Mb-ME can attenuate skin inflammation caused by UVB and reduce the likelihood of skin cancer that may accompany it.

Furthermore, hyaluronan synthases (HAS-1, HAS-2 and HAS-3) synthesized hyaluronan, the key molecule involved in skin moisture [38]. Hyaluronan is a glycosaminoglycan found naturally in the skin that helps binding moisture to collagen, making it plumper and more hydrated [36]. Hyaluronidases (HYALs) cleave high molecular weight hyaluronan into smaller fragments following further degradation [39]. FLG and TGM contribute to the formation of cornified cell envelopes, a structure that forms a protective barrier from water loss and allergens [40]. Therefore, FLG and TGM are essential molecules in epidermal terminal differentiation and skin barrier function. Our results demonstrated that Mb-ME upregulated mRNA expressions of FLG, TGM, and HASs, suggesting great ability of Mb-ME to increase skin moisturizing conditions under normal treatment. On the other hand, UVB irradiation and H_2_O_2_ treatment increased the expression of HYALs and exacerbated the cleavage of hyaluronan, leading to the loss of water. Intriguingly, Mb-ME inhibited production of HYAL-1, -2 and -4, indicating that it can relieve the loss of water under UVB or H_2_O_2_ stimulation. Mb-ME exerted the ability to drastically increase skin moisture and promote the formation of the outer layer of the skin. Taken together, the above results indicate that Mb-ME manifests multiple roles of protecting the skin from ECM degradation, loss of moisture, and inflammation.

Consistent with previous reports [41], UVB triggered ROS production and DNA damage, while they were abolished by Mb-ME treatment using DCF-DA staining as well as DAPI staining, respectively. These results show Mb-ME can repair DNA breaks and attenuates oxidative stress. Accumulated evidence suggests that UVB irradiation stimulates the apoptosis of skin cells [42]. Similar results were observed through the quantification of apoptotic cells by flow cytometry. The effect of Mb-ME on apoptosis was accordant with its recovery capacity of HaCaT cell viability upon UVB exposure. This indicates that Mb-ME can decrease apoptotic cells caused by UVB.

Caspases are the core executive components of both extrinsic and intrinsic apoptosis. At least 14 Caspases have been identified so far [43]. They are generally divided into two classes: initiator caspases (caspase-2, -8, -9 and -10) and executioner caspases (caspase-3, -6 and -7) [44,45]. Activated caspases cleave a wide range of molecular targets, ultimately leading to cell death [43]. As shown in Figure 5A–C, caspases were activated in response to UVB stimulation, whereas UVB-enhanced apoptosis was abolished by Mb-ME. Our study thus implied Mb-ME exerts its anti-apoptotic effect through caspases.

Moreover, Sirt1 (Sirtuin Type 1), also known as one member of class III histone deacetylases, is a protein involved in apoptosis, cell survival, aging, and glucose homeostasis [46]. The decline of the Sirt1 gene after UVB irradiation was drastically hindered by Mb-ME (Figure 5D). The mRNA expression of p53, a cellular target of Sirt1, was enhanced by UVB exposure whereas the elevation was reduced by Mb-ME (especially at 100 μg/mL). These data findings suggested that Mb-ME suppressed cellular senescence and prolonged its lifespan. However, Mb-ME only slightly reduced melanin contents and secretion without the involvement of tyrosinase. The weak effect of Mb-ME on melanin production may be caused due to that some ingredients of Mb-ME can only control the functional involvement of the structural proteins of melanosomes or the proteins required for melanin transport and distribution. Since current data are not enough to make putative interpretation, further study should be conducted for this.

To explore the role of Mb-ME in molecular signaling, we first studied AP-1 and NF-κB because they are transcription factors that are activated upon UVB irradiation. The augmentation of AP-1 and NF-κB increased the production of cytokines, chemokines, and several acute phase proteins. Constant skin exposure to UVB can lead to chronic inflammation, eventually leading to skin disease including psoriasis, atopic dermatitis, and cancer [47]. Our results strongly demonstrated that Mb-ME not only suppressed the subunits of AP-1 and NF-κB but inhibited their upstream molecules after UVB stimulation.

## 4. Materials and Methods

### 4.1. Materials

Neonatal, primary HDF cells (a human dermal fibroblast cell line) were obtained from Lifeline (Oceanside, CA, USA). HaCaT (a human keratinocyte cell line), HEK293 (a human embryonic kidney 293 cell line), and B16F10 cells (a murine melanoma cell line) were purchased from the American Type Culture Collection (Rockville, MD, USA). Dulbecco’s modified Eagle’s medium (DMEM), penicillin, streptomycin, phosphate-buffered saline (PBS), and fetal bovine serum (FBS) were purchased from HyClone (Grand Island, NY, USA). Mushroom tyrosinase, polyethylenimine (PEI), H_2_DCF-DA, Kojic acid, arbutin, SB203580, SP600125, α-melanocyte stimulating hormone, U0126, BAY11-7082, and Annexin V-FITC Apoptosis Detection Kit were obtained from Sigma-Aldrich (St. Louis, MO, USA). Specific antibodies were obtained from Cell Signaling Technology (Beverly, MA, USA) or Santa Cruz Biotechnology (Santa Cruz, CA, USA).

### 4.2. Plant Material and Extract Preparation

The collection and identification of *M. baccata* (L.) Borkh. was performed as previously described paper [48] by some modification as follow. All solvents were of HPLC grade (Merck, Germany). The leaves and shoots of *M. baccata* were collected, dried by lyophilization and then ground into a fine powder. Of the powder, 19 g was sonicated in 1 L of 99.9%(*v/v*) methanol for 15 min in an ultrasound bath at 45 °C and resting for 2 h [48]. After filtration with non-fluorescence cottons, the residue was extracted again for another two times with fresh methanol following same process. *M. baccata* extract was obtained by drying and concentrating all the resultant products under reduced pressure at 45 °C using a rotary evaporator. After freeze-drying, 4.09 g (21.5%) *M. baccata* extract was produced [48]. Before treatment, Mb-ME, in powder form, was dissolved in 100% DMSO and stored at −20 °C until use. Mb-ME was further diluted to working concentrations in cell culture medium.

### 4.3. Cell Culture

HaCaT and B16F10 cells were cultured in 10% FBS containing DMEM medium and 1% antibiotics. HEK293 and HDF cells were cultured in DMEM medium supplemented with 5% FBS and 1% antibiotics [49]. The cells were maintained in a 5% CO_2_ incubator at 37 °C [50].

### 4.4. Cell Viability Assay

HaCaT, HEK293, HDF, and B16F10 cells were plated into 96-well plates. B16F10 cells were treated with Mb-ME for 48 h, while other cells were incubated with Mb-ME for 24 h. In cases of UVB and H_2_O_2_ induction, the cells were treated with Mb-ME for 30 min, stimulated by UVB or H_2_O_2_, and further treated with Mb-ME for 24 h [48]. Conventional MTT assay was conducted to investigate cell viability [51]. 10 μL MTT solution was aliquoted to each well. After 3 h reaction, 100 μL MTT stopping solution was distributed to each well. Absorbance at 570 nm was detected.

### 4.5. DPPH Assay

The free radical scavenging ability of Mb-ME was tested by the DPPH radical scavenging assay as previous described [52]. DPPH (250 μM) was incubated with either Mb-ME (25–200 μg/mL) or ascorbic acid (500 μM) for 30 min at 37 °C. The DPPH scavenging ability was investigated by measuring absorbance at 517 nm.

### 4.6. ABTS Assay

ABTS radical cations were generated by mixing ABTS and potassium sulfate (1:1) overnight at room temperature [17]. Mb-ME (6.25–200 μg/mL) or ascorbic acid (500 μM) was reacted with ABTS solution for 30 min at 37 °C [41]. The ABTS scavenging capacity was measured by determining the absorbance at 730 nm.

### 4.7. UVB and H_2_O_2_ Treatment

HaCaT or HDF cells were treated with Mb-ME for 30 min, exposed to UVB irradiation (30 mJ/cm^2^, Bio-Link BLX-312, Vilber Lourmat, France), incubated with Mb-ME for 24 h [52]. Mb-ME was used to treat HaCaT or HDF cells for 30 min and H_2_O_2_ (500 μM) was employed to induce the cells for 24 h [53].

### 4.8. ROS Generation

HaCaT cells were seeded in 12-well plates overnight. Cells were pretreated with Mb-ME for 30 min and then irradiated with UVB in the absence or presence of Mb-ME for 24 h [54]. After harvesting and washing with PBS, cells were resuspended in 300 μL DCF-DA and incubated for 30 min in the dark [55,56]. The samples were analyzed by flow cytometry using a 485-nm laser for excitation and a 535-nm laser for emission. 

### 4.9. Gas Chromatography-Mass Spectrometry

A gas chromatography–mass spectrometry (GC–MS) instrument was used to analyze the active composition from the methanol extract of *M. baccata*. GC-MS analysis of Mb-ME was performed by the Cooperative Center for Research Facilities at Sungkyunkwan University. GC-MS conditions were as follows: Agilent 8890 GC (Santa Clara, CA, USA), Agilent J&W DB-624 Ultra Inert GC column (60 m  ×  0.25 mm, 1.40-μm film thickness); injector temperature: 220 °C; temperature program: shrink temperature was 35 °C for 4 min, the temperature rose at a gradient of 8 °C/min up to 180 °C for 4 min, then at 15 °C/min to 230 °C for 1 min [57]. Ion source temperature was 200 °C. Carrier gas: Helium (1.2 mL/min), Sample injection volume: 2 μL, Ionization energy: 70 eV [57]. To identify unknown phytochemicals in Mb-ME, known phytochemicals from National Institute of Standards and Technology (NIST) library were used as comparison criteria.

### 4.10. Semi-Quantitative RT-PCR

HaCaT or HDF cells were exposed to UVB or H_2_O_2_ and incubated with Mb-ME for 24 h. B16F10 cells were induced with α-MSH and incubated with Mb-ME or arbutin for 48 h [58]. To investigate the effect of Mb-ME on moisture and collagen, retinol or Mb-ME were used to treat HaCaT cells for 24 h in the absence or presence of inducer (UVB and H_2_O_2_). To prove the effect of Mb-ME on the signaling pathway, HaCaT cells were induced by UVB and treated with MAPKs inhibitors (U0126, SP600125, and SB203580) or NF-κB inhibitors (BAY11-7082). Total mRNA was extracted from the treated cells and complementary DNA was synthesized as reported previously [59]. Primer sequences are listed in Table 2 and Table 3.

### 4.11. PI and Annexin V-FITC Staining

HaCaT cells treated with UVB and Mb-ME were harvested, washed with cold PBS twice, and resuspended in 1× binding buffer containing 100 mM HEPES, 140 mM NaCl, and 25 mM CaCl_2_, pH 7.4. Propidium iodide (PI, 5 μL) and Annexin V-FITC (5 μL) staining solution were distributed to each sample, and the samples were kept in the dark for 15 min. Each tube was aliquoted with 1× binding buffer (400 mL) and immediately analyzed by flow cytometry [60,61].

### 4.12. DAPI Staining

Mb-ME was used to treat HaCaT cell for 30 min and H_2_O_2_ was employed to stimulate cells for 24 h. Cells were then washed with PBS twice and fixed with 3.7% paraformaldehyde for 15 min [62]. Then, cells were washed with PBS twice and stained with DAPI reagent for 30 min in the dark [58]. The staining solution was discarded, and cells were washed three times by PBS. Images of the cells were captured using a fluorescence microscope.

### 4.13. Plasmid Transfection and Luciferase Reporter Assay

HEK293 cells were transiently transfected with Col1A1-Luc and β-galactosidase plasmids for 24 h using PEI [63]. Mb-ME or retinol were employed to treat cells for an additional 24 h. Luciferase assay was subsequently conducted.

### 4.14. Protein Lysates Preparation and Immunoblotting

HaCaT cells were irradiated with UVB with or without Mb-ME for 24 h. Cells were harvested, washed by PBS and lysed in lysis buffer [50 mM Tris-HCl (pH 7.5), 120 mM NaCl, 2% NP-40, 25 mM b-glycerophosphate (pH 7.5), 20 mM NaF, and protease inhibitor cocktails]. Total lysates were centrifuged at 12,000 rpm for 5 min. The protein concentration of the samples was measured by Bradford assay [64]. The proteins were then subjected to immunoblotting. Phospho- or total levels of pro- and cleaved caspases, c-Fos, p65, ERK, JNK, p38, PI3K, AKT, PDK1, IKKα/β, IκBα and β-actin were detected [65].

### 4.15. Tyrosinase Activity Assay

L-dopamine (6 mM), kojic acid, mushroom tyrosinase (100 units/mL), or indicated concentrations of Mb-ME were mixed for 15 min in 96 well plate at room temperature [66]. The absorbance at 475 nm was performed to evaluate the tyrosinase activity.

### 4.16. Melanin Formation and Secretion Analysis

In order to test melanin secretion, B16F10 cells were grown in 96 well plates and treated with Mb-ME, α-MSH, or arbutin for 48 h [67]. 100 μL cell culture fluid was transferred to a new 96 well plate. The absorbance of media at 475 nm was detected to measure secreted melanin by B16F10 cells. For melanin content analysis, the cells were lysed in lysis buffer and centrifuged at 12,000 rpm for 5 min. The pellets were then dissolved in dissolving buffer (10% DMSO in 1 M NaOH) at 55 °C for 30 min [68]. The absorbance of the solution was determined at 405 nm.

### 4.17. Statistical Analysis

All results are expressed as means ± standard deviations of at least triplicate independent experiments. All data were analyzed using the Kruskal–Wallis/Mann–Whitney test. All statistical comparisons were performed using SPSS software. *p* < 0.05 was considered statistically significant.

## 5. Conclusions

**Figure 8:**
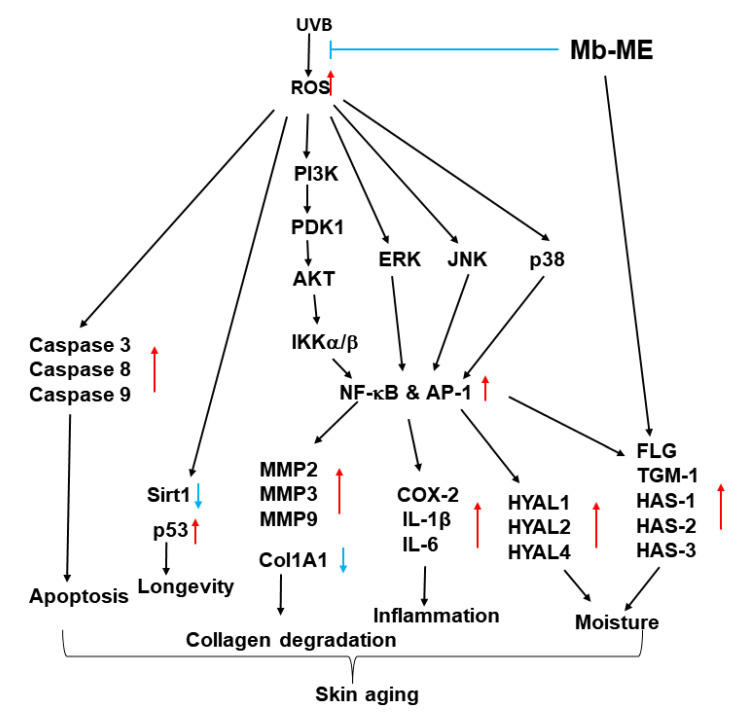
The mechanism by which Mb-ME exerts anti-aging and anti-oxidative effects.

In this study, we attempted to summarize the function and mechanism of Mb-ME on aging, moisture retention, apoptosis, and antioxidant capacity. Mb-ME not only protected HaCaT cells from UVB-induced ROS and apoptosis, but also suppressed the phosphorylation of MAP kinases as well as NF-κB signaling. Mb-ME abolished the production of MMPs, p53, inflammatory genes, and HYALs, while restoring the expressions of Col1A1, Sirt1, and HASs. The schematic description of the mechanism is shown in Figure 8. Our results demonstrated that Mb-ME may be a potential therapeutic candidate for the prevention of skin aging. Since protective activity of Mb-ME against UVB irradiation under its pretreatment conditions in skin cells is promising, we are hoping to develop Mb-ME as a major ingredient of new suncream product. For this, additional experimental approaches such as formulation work and clinical tests will be followed.

## Figures and Tables

**Table 1:** GC-Mass results of methanol extract of *Malus baccata*.

Peak No.	R.T. (min)	Compound Name	Peak Area %
1	8.500	2-Propanone	4.212
2	10.125	1-Hydroxy-2-butanone, 2-Propenoic acid, 2-hydroxyethyl ester	2.007
3	10.499	Hexanal, Butanal	0.531
4	10.625	1-Propanol,3-Isopropoxy alanine, Butyric acid hydrazide	0.868
5	10.780	Di-n-propyl ether, Propanoic acid, Methyl ester	1.038
6	11.696	Furfural	0.401
7	12.192	2-Furanmethanol	1.597
8	13.797	2-Heptenal, (Z)-2-Heptenal, (E)-Cyclopentane	0.732
9	13.959	2,4-Dihydroxy-2,5-dimethyl-3(2H)-furan-3-one	0.557
10	15.074	Phenol	5.569
11	15.755	2-Butylamine, N-pentyl-Thiophene-3	1.723
12	16.413	1,3,5-Triazine-2,4,6-triamine,monoamide,propargyl ester	7.152
13	17.717	4H-Pyran-4-one, 2,3-dihydro-3,5-dihydroxy-6-methyl-	5.802
14	18.106	Methyl salicylate	7.156
15	19.104	Benzofuran	20.304
16	19.551	5-Hydroxymethylfurfural	6.290
17	20.092	2-Methoxy-4-vinylphenol	3.622
18	20.512	Hydroquinone, Isosorbide	11.142
19	25.288	N-m-Tolyl-succinamic acid, 2-Butanone, 4-(4-hydroxyphenyl)-Acetamide	4.232
20	27.565	Benzeneacetic acid,4-hydroxy-Benzeneacetic acid, methyl ester	9.241
21	28.281	piperidine, 4-(4-methoxyphenoxy)-	4.235

**Table 2:** Human primer sequences used in this study.

Gene Name		Sequencing (5′ to 3′)
FLG	F	AGGGAAGATCCAAGAGCCCA
	R	ACTCTGGATCCCCTACGCTT
MMP-3	F	ATCCTACTGTTGCTGTGCGT
	R	CATCACCTCCAGAGTGTCGG
HAS-3	F	GTTCGCGGCTGCTTTGAC
	R	GTAGCCCGTCACATAGGCTG
GAPDH	F	GGTCACCAGGGCTGCTTTTA
	R	GATGGCATGGACTGTGGTCA
Sirt1	F	CAGTGTCATGGTTCCTTTGC
	R	CACCGAGGAACTACCTGAT
HAS-1	F	CCACCCAGTACAGCGTCAAC
	R	CATGGTGCTTCTGTCGCTCT
COX-2	F	CAAAAGCTGGGAAGCCTTCT
	R	CCATCCTTCAAAAGGCGCAG
MMP-2	F	ACGACCGCGACAAGAAGTAT
	R	CTGCAAAGAACACAGCCTTCTC
TGM1	F	GAAATGCGGCAGATGACGAC
	R	AACTCCCCAGCGTCTGATTG
IL-6	F	TTCGGTCCAGTTGCCTTCTCC
	R	TGAGGTGCCCATGCTACATTT
HYAL-2	F	TACACCACAAGCACGGAGAC
	R	ATGCAGGAAGGTACTGGCAC
MMP-9	F	CAACATCACCTATTGGATCC
	R	CGGGTGTAGAGTCTCTCGCT
HYAL-1	F	CAGAATGCCAGCCTGATTGC
	R	CCGGTGTAGTTGGGGCTTAG
p53	F	CAGCCAAGTCTGTGACTTGCACGTAC
	R	CTATGTCGAAAAGTGTTTCTGTCATC
HYAL-4	F	TGAGCTCTCTTGGCTCTGGA
	R	AGGCAGCACTTTCTCCTATGG
COL1A1	F	CAGGTACCATGACCGAGACG
	R	AGCACCATCATTTCCACGAG
IL-1β	F	TGAGCTCGCCAGTGAAATGA
	R	AACACGCAGGACAGGTACAG
HAS-2	F	TTCTTTATGTGACTCATCTGTCTCACCGG
	R	ATTGTTGGCTACCAGTTTATCCAAACG

**Table 3:** Mouse primer sequences used in this study.

Gene Name		Sequencing (5′ to 3′)
MITF	F	GGGAGCTCACAGCGTGTATT
	R	CTAGCCTGCATCTCCAGCTC
TYRP1	F	GTGAGCAGCTCTGTGCTGTA
	R	AGGGGGAGGACGTTGTAAGA
Tyrosinase	F	GGCCAGCTTTCAGGCAGAGG
	R	TGGTGCTTCATGGGCAAAAT
GAPDH	F	ACCCTTAAGAGGGATGCTGC
	R	GTTCACACCGACCTTCACCA

## Data Availability

The data is contained within the article.

## Abbreviations

Mb-ME	*Malus baccata* methanol extract
AP-1	Activator protein 1
NF-κB	Nuclear factor-κB
ROS	Reactive oxygen species
MMP	Matrix metalloproteinases
RT-PCR	Reverse transcription polymerase chain reaction
IL	Interleukin
HA	Hyaluronan or hyaluronic acid
HYAL	Hyaluronidases
COX_2_	Cyclooxygenase
MAPK	Mitogen-activated protein kinases
α-MSH	α-Melanocyte-stimulating hormone
ECM	Extracellular matrix
